# Update on the Improving Access to Psychological Therapies programme in England: author's reply^†^

**DOI:** 10.1192/pb.bp.115.052399

**Published:** 2015-10

**Authors:** Sami Timimi

**Affiliations:** 1 Horizon Centre, Lincoln

## Abstract

Fonagy & Clark confirm in their rebuttal that they have an ideological commitment to the failed technical model of understanding and intervening in mental health problems that dominates current service provision. They fail to acknowledge the limitations and problems associated with Improving Access to Psychological Therapies (IAPT) and Children's and Young People's IAPT (CYP-IAPT) and offer an unconvincing explanation for why they did not allow some of the vast tax payers' money that they had at their dispoal to be used to implement evidence supported relational models.

I will mainly comment on professors Fonagy and Clark's article^1^ as they have attempted an evidence-supported rebuttal. Dr Law's letter^2^ calls for more dialogue, which by itself will not change the implementation fundamentals, whereas Ms Swaile's letter^3^ points out the obvious – that if you spend more on psychological therapies, more of them will be available – whereas my editorial^4^ was critiquing how this extra available money was spent.

Fonagy & Clark's article reminded me why I initially got excited about Children and Young People's Improving Access to Psychological Therapies (CYP-IAPT), with its desire to engage with the evidence and talk of improving access, collaborative working, focusing on outcomes, local learning and so on. My original enthusiasm was misplaced as there is a fatal flaw, which they seem unable to get past – their belief that the poor outcomes and inefficiencies of mainstream services happen because we are not rigorous enough in the way we enforce technical aspects of care. Their ideological intransigence on this matter has created a cartel-like monopoly where existing and successful alternative models have no opportunity to be tried.

Their critique of relational/contextual models (they refer to as ‘common factors’) is unconvincing. The most rigorous meta-analyses of evidence from randomised controlled trials (RCTs) note that of course papers can be found showing some model differences, but the overall evidence base finds that across presentations by far the biggest factors influencing outcomes have little to do with what we do – so called extra-therapeutic factors (the real-life contexts, beliefs and histories), whereas within therapy it is the therapeutic alliance.^5^ Therapeutic alliance is not a one-dimensional construct and includes, for example, the degree of ‘engagement’ – a two-way process including understanding what is meaningful to the patient. Thus, if a computer program provides a meaningful methodology for a patient, then that is where their ‘alliance’ may form.

The authors predictably avoid bigger issues with National Institute for Health and Care Excellence (NICE)-guideline-derived evidence-based-therapies (EBTs). In mental health (unlike the rest of medicine), NICE guidelines are eminence not evidence based, in other words they rely more on who was on the guideline group than what the evidence says.^6,7^ NICE guidelines focus on process adherence, but have little to say about outcomes. They derive from diagnostic constructs that have done little to advance scientific knowledge or clinical practice, and have no capacity to match treatments to aetiology, thus failing the basics required of a technical model.^8^ Mental health treatment RCTs use exclusion criteria, which often means the sort of multi-problem, diagnostic overlap patients typical of those who attend our clinics are not adequately represented. Like me, Fonagy & Clark want to do something about the dreadful record for outcomes that real-world mental health services have. Their solution is to ‘beef up’ existing diagnosis-based NICE-guideline EBTs that we have been using for years, but using more manualised process adherence. But the fantasy that expertise in technique is king is what got us into this mess in the first place. This ideological commitment seems to have by-passed simple logic. If the outcomes with a patient show improvement, does it matter what model you use to help achieve this? If an outcome is not improving, then it surely does matter and irrespective of what your manual says you may need a rethink what you are doing altogether.

As far as their concept of ‘relational’ is concerned, they describe a collaborative ‘light’ model. In the primacy of the technical model, ‘collaborative’ essentially amounts to convincing the patient that the expert knows what is wrong with them and what the right treatment is. Prioritising the relational means that therapy is potentially ‘co-constructed’ at every step. In a proper relational model we are constantly encountering ‘experts by experience’ whose insights, skills, choices, autonomy and resources should be harnessed to help shape therapy session by session.

Fonagy & Clark's article does little to dent the critique that CYP-IAPT has not, thus far, managed to develop patient-empowering, outcomes-focused, collaborative practice. Tellingly, after 4 years of implementation they were unable to reference any patient outcome data for CYP-IAPT. In the 3 years of our local Outcomes Orientated Child and Adolescent Mental Health Services (OO-CAMHS) project, we have amassed a database with over 4000 discharged cases with outcome ratings where a reliable clinical improvement and/or ‘recovered’ rate of 75% is being recorded. It is time for CYP-IAPT to stop being frightened of relational models and give them a seat at their table. If Fonagy & Clark wish to maximise the chances of having services that can improve the lives of many more people, then they should embrace the opportunity to include alternative models such as the Partners for Change Outcome Management System/OO-CAMHS that have a proven track record in real-world services, to keep open possibilities for discovering ways of designing services that are most effective and efficient. If they remain ideologically belligerent (as they have thus far), every word of my critique stands.
